# HoMEcare aRm rehabiLItatioN (MERLIN): telerehabilitation using an unactuated device based on serious games improves the upper limb function in chronic stroke

**DOI:** 10.1186/s12984-021-00841-3

**Published:** 2021-03-16

**Authors:** Samantha G. Rozevink, Corry K. van der Sluis, Ainara Garzo, Thierry Keller, Juha M. Hijmans

**Affiliations:** 1 Department of Rehabilitation Medicine, University of Groningen, University Medical Center Groningen, Groningen, The Netherlands; 2 Neurorehabilitation Area, Health Division of TECNALIA, Basque Research and Technology Alliance (BRTA), San Sebastian, Spain

**Keywords:** Stroke, Rehabilitation, Telerehabilitation, Home training, Upper limb, Hand, Training device, Task specific

## Abstract

**Background:**

HoMEcare aRm rehabiLItatioN (MERLIN) is an unactuated version of the robotic device ArmAssist combined with a telecare platform. Stroke patients are able to train the upper limb function using serious games at home. The aim of this study is to investigate the effect of MERLIN training on the upper limb function of patients with unilateral upper limb paresis in the chronic phase of stroke (> 6 months post stroke).

**Methods:**

Patients trained task specific serious games for three hours per week during six weeks using an unactuated version of a robotic device. Progress was monitored and game settings were tailored through telerehabilitation. Measurements were performed six weeks pre-intervention (T0), at the start (T1), end (T2) and six weeks post-intervention (T3). Primary outcome was the Wolf Motor Function Test (WMFT). Secondary outcomes were other arm function tests, quality of life, user satisfaction and motivation.

**Results:**

Twelve patients were included, ten completed the training. From start of the intervention to six weeks follow up, WMFT improved significantly with 3.8 points (p = .006), which is also clinically relevant. No significant changes in quality of life were observed. Patients were overall satisfied with the usability of the device. Comfort and the robustness of the system need further improvements.

**Conclusion:**

Patients in the chronic phase of stroke significantly improved their upper limb function with the MERLIN training at home.

*Trial registration* This study is registered at the Netherlands Trial Register (NL7535). Registered 18–02-2019, https://www.trialregister.nl/trial/7535.

## Background

Stroke is common in the general population, with annually 1.1 million people in Europe suffering from stroke [1]. After stroke, almost 77% of the patients have a paretic upper limb with loss of function, leading to a lower quality of life [2, 3]. During recovery, patients and therapists have to focus on many rehabilitation goals such as improving balance and gait, and reducing spasticity. Upper limb function is often undertreated according to patients, and consequently many return home from a hospital or rehabilitation center with remaining disabilities [4, 5]. It is generally believed that after six months, a plateau in functional motor recovery is reached [6–8]. However, more evidence is emerging that patients in the chronic phase of stroke are able to improve the upper limb function significantly [7, 8]. Therefore, training has to involve a few key parameters that are important for motor learning such as high intensity training, many repetitions and a task specific approach [9]. However, this is not yet generally available in practice for patients in the chronic phase of stroke, possibly due to the high costs of individual sessions with a therapist. More affordable ways of providing intensive, task specific therapy in the chronic phase of stroke are needed.

Home rehabilitation could fill the gap of insufficient amount of training for patients in the chronic phase of stroke. Using telerehabilitation (providing rehabilitation via online communication), the patient can train at home, and the therapist is able to provide assistance from a distance using online communication. Telerehabilitation combined with serious games may increase the engagement of the patient with the therapy [10]. Serious games are computer games used for training or educational purposes [11]. Keeping patients motivated is of utmost importance because patients express that performing the same exercises may lead to boredom and inhibits continuation of training [5]. By providing various serious games with different levels of difficulty, the patient may be more motivated to continue the training program, which may lead to more improvement in arm function [12]. A positive dose–response relation between the amount of training and upper limb improvement emphasizes the need for intensive training [13].

Serious games and telerehabilitation can be combined with training devices to even further increase the effectiveness of therapy. Robot-assisted training devices, containing an actuator or motor, have shown to be effective in improving the upper limb function in stroke patients [14]. However, these devices are often expensive and therefore not suitable to be used at home and supervision is needed due to safety issues. Unactuated devices could be a cheaper alternative, safer to use at home and may have the same training effect. In comparison to the large amount of robotic devices that are studied, only a few studies investigated non-robotic devices. The Therapy Wilmington Robotic Exoskeleton (T-WREX) and its commercially available version Armeo Spring are the most investigated non-robotic devices that were successful in improving the arm function [15–17]. Although the name of T-WREX presumes a robotic device, both T-WREX and Armeo Spring are passive arm orthoses based on springs which contain several sensors to measure movements of the arm and hand. Unfortunately, these devices are still expensive due to mechanical complexity and too large to be used at home. According to therapists, costs and home use are important determinants for successful rehabilitation devices [18]. The Sensorimotor Active Rehabilitation Training (SMART) Arm is a non-robotic device which has been used at home during a case study to train reaching movements [19, 20]. Preliminary results looked promising, however the device is large and heavy and assistance from another person is needed before starting the training. It can be concluded that home rehabilitation with non-robotic devices is still in its infancy. A solution for a compact, affordable device that allows stroke patients to train at home is proposed.

HoMEcare aRm rehabiLItatioN (MERLIN) was designed to provide upper limb training at home. MERLIN is a combination of two existing rehabilitation solutions: ArmAssist system, a robotic device based on serious games with visual feedback of the training (TECNALIA R&I, Spain) [21, 22], and the Antari Homecare telerecare platform (GMV, Spain) [23]. The ArmAssist system is a portable system that can measure the patient’s active or passive movements. A previous study demonstrated that deploying of the motorized version of the ArmAssist system early after stroke in a rehabilitation center was safe and more effective in comparison to intensive therapist-guided conventional therapy [24]. In the MERLIN project, the ArmAssist system was used in its non-motorized version, which implied that only the active movements of the patient were measured. The unactuated version of the device was selected for home use because it was considered safer when constant professional supervision is not possible. In combination with a telecare platform, the ArmAssist system including the serious games seems suitable for home training.

The aim of the study was to investigate if MERLIN training can improve the arm function of chronic stroke patients during a 6-week task specific home rehabilitation program. We hypothesized that there will be a clinically relevant improvement on the WMFT after six weeks of MERLIN training. Gross motor movements, such as shoulder flexion and elbow flexion, are expected to improve the most after MERLIN training. The WMFT contains many items to test gross motor movement and is therefore the most suited outcome measure to answer this research question. Secondary aim was to investigate the quality of life, usability and patient motivation.

## Methods

### Sample size

The previous study with ArmAssist system in subacute stroke showed a large effect size of 0.95 for the Wolf Motor Function Test (WMFT) [24]. In this study we included patients in the chronic phase of stroke, we therefore expected a medium effect size. With a power of 0.8, alpha of 0.05, and partial eta squared of 0.12, the sample size was calculated to be 12 participants for this repeated measures study. Including a drop-out rate of 20%, we aimed to include 15 patients.

### Subjects

Twelve participants were enrolled in the study between August 2019 and January 2020 (Table 1). The inclusion criteria were: 1) ≥ 18 years; 2) first incidence of a clinical single stroke or stuttering stroke with unilateral hemiparesis; 3) > 6 months and < 3 years post-stroke; 4) score on the Fugl-Meyer Assessment–Upper Extremity (FMA-UE) motor function of < 50; 5) able to perform finger extension three times and have some proximal voluntary movement capability in the arm; 6) ability to give informed consent, understand and execute simple instructions; 7) visual, mental and cognitive ability to assimilate and actively participate in the protocol; 8) being able to speak and understand Dutch or English; 9) know how to operate a computer (or have someone available for assistance) and have the possibility to train at home (having room to set up the system and access to Wi-Fi). Exclusion criteria were: 1) Depression (score four or higher on the EuroQoL-5D (EQ-5D) depression item); 2) rheumatologic, orthopaedic or other neurological disorders of the upper arm; 3) receiving occupational therapy or physiotherapy specifically focusing on the arm/hand function.

**Table 1:** Participant characteristics (N = 12)

	Mean ± SD
Age (Years)	64.8 ± 8.5
Gender (Male/Female)	8/4
Time since stroke (Months)	22.9 ± 9.3
Stroke type (Hem/Isch)	1/11
Side hemiparesis (Left/Right)	7/5
Dominant side (Left/Right)	0/12
Social status	
Married	10
Divorced	1
Living together	1
Education	
Primary school	2
Pre-vocational education	6
Higher professional education	4
Work	
Did not work before stroke	1
Stopped after stroke	5
Working	0
Retired	4
Other	2
Comorbidities	
Diabetes Mellitus	1
Sleeping disorder	1
COPD	2
Cancer	1
Other	4

*COPD* Chronic Obstructive Pulmonary Disease, *Hem* Haemorrhagic, *Isch* Ischemic, *SD* standard deviation

### Design

A repeated measures within subject design, where the participant was their own control, was conducted. A control period was included in which participants did not follow any functional upper limb training. This design was chosen to take natural recovery into account, although this was not expected due to the chronic phase of stroke. Participants were recruited via an information letter sent by rehabilitation physicians of two rehabilitation centers: University Medical Center Groningen (UMCG) and Rehabilitation Friesland, both located in the northern part of the Netherlands. Furthermore, informative presentations were given at local stroke peer groups. Participants who showed interest in the study by returning the participation form, were contacted by phone to check the initial eligibility criteria. If patients seemed eligible, an intake procedure was performed at the patient’s home to confirm the FMA-UE and EQ-5D health state. Participants had to provide written informed consent before the intake procedure took place.

### Intervention

According to Kwakkel et al., the minimal training time needed for significant improvement of the upper limb function is 16 h [25], therefore participants were instructed to train at home for at least 18 h (16 + 10%), divided into three hours per week, over a course of six weeks. Therapy compliance was assessed by the telerehabilitation platform since, amongst other variables, completion of the games was registered.

### Device hardware

**Fig. 1:**
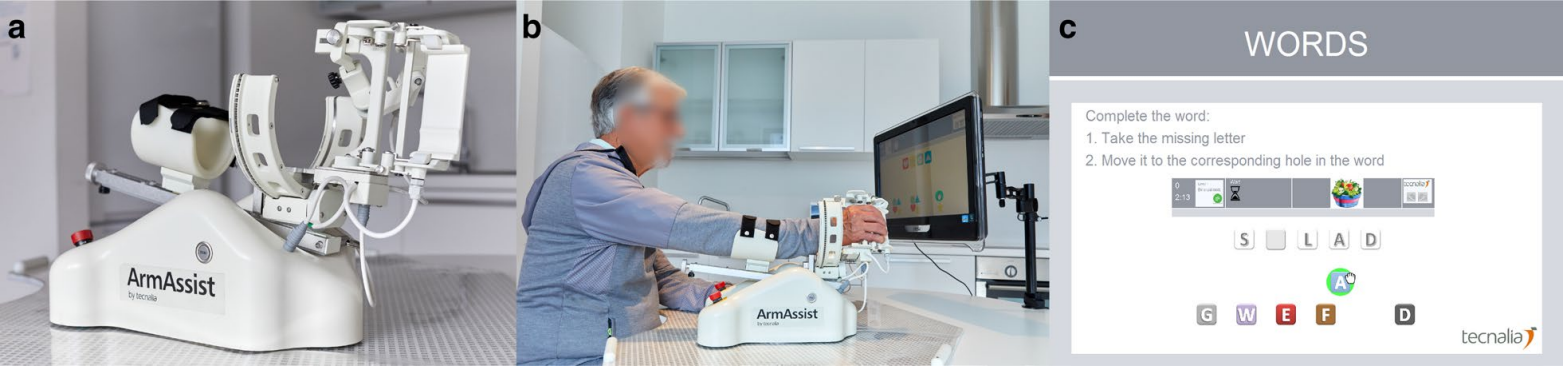
** a** ArmAssist device; **b** MERLIN system for training at home; **c** Example of the game “Words”

Participants were equipped with a placemat, ArmAssist robotic device and a tablet (portable computer) with the MERLIN telerehabilitation software, which included the serious games. The ArmAssist (Fig. 1a) is a low-cost passive device which assesses the participant’s arm and hand movements and represents it on a computer screen similar to a computer mouse. The ArmAssist contains a camera and encoders on the wheels to measure the absolute position and orientation of the device on the placemat, a potentiometer to measure wrist angle, a load cell to measure vertical force, and Force Sensing Resistor sensors for measuring the grasping force. This assembly of sensors represents the movements in the games accordingly. Different movements can be performed to interact with the games: moving freely in three different degrees over the table (horizontal, vertical and rotation), grasping/releasing, wrist rotation and isometric lifting (measuring the arm weight on the device). The gravity support makes it easier to move the arm in space [26]. The patient is able to train ab-/adduction and ante-/retroflexion in the shoulder, elbow flexion/extension, wrist pro-/supination, mass flexion/extension of the fingers and thumb separately and muscle strength. A total of seven degrees of freedom (DoF) were available: three DoF for the position and orientation of the device, one DoF for the lifting force and three DoF on the hand add-on to perform finger and thumb movements and wrist rotation. The movements that were performed with the ArmAssist device were used to interact with the serious games that were visualized on the computer screen (Fig. 1b). For example, the participant was asked to turn a card in a memory game (find two cards with the same picture) by pro-/supinating the wrist (“Memory game”) or grasp a virtual written letter to complete a word that was depicted with missing letters (“Words game”, Fig. 1c). Another example is “in the bar” game, where the participant is challenged to pick up a virtual bottle by performing a grasping motion and upward lifting force. The bottle needs to be emptied in a glass by performing a pronation while maintaining the grasp.

### Device software

Every day, six games of five minutes each were planned for each participant by the researcher through the telemonitoring system, summing to at least 30 min of practice. It was advised to complete 30 min of training six days a week, resulting in at least three hours training per week as was prescribed. Participants could however adapt the training duration and frequency according to their preferences. The therapy was task specific, repetitive, intensive and randomly ordered (position of objects in the games was random) [9]. Feedback was provided by a score on the games and visual feedback provided at the computer screen. The therapy program was designed and remotely adjusted to the participants’ individual needs by the researcher, who had weekly consultations with an independent experienced occupational therapist. The first week of training was mainly used to familiarize the participant with the device using simple games. In the subsequent weeks the training increased in difficulty by adding more movements and combinations of movements (e.g. grasping, lifting and moving the device). If the patient had trained, the therapist was able to see time of training, score, level, and patient’s messages.

Once every 2 weeks, the researcher visited the participants at home to recalibrate the system to the range of motion they were able to reach. With the calibration of the settings, the games continued to be challenging but achievable for the participant, since they were adjusted to 80% of the maximum range. Calibration could be performed without a researcher/therapist, but to ensure that the calibration was performed consistently during the study, all calibrations were supervised by the researcher.

### Outcomes

Three commonly used arm function tests were assessed to answer the research questions. The primary outcome was the WMFT to test the upper limb motor ability of the participant. The WMFT has two subscales: the Functional Ability Scale (FAS) and the time score per item. The FAS is used to score the items of the WMFT on a 6 point scale (range 0–5, maximum 75 means normal motor function). Maximum mean time score was 120 s, less time to complete the item indicated better movement performance. The WMFT validity and reliability has been found to be excellent [27–31]. Intraclass Correlation Coefficient for inter-rater and intra-rater reliability were 0.94 and 0.95, respectively [28]. The Minimal Clinically Important Difference (MCID), a measure for smallest change that is meaningful to the patient, is between 3 and 6 points on the FAS and 1.5 and 2 s for the time domain, calculated from an anchor based and distribution based method, respectively [32].

Secondary arm function outcomes were the FMA-UE and Action Research Arm Test (ARAT). The FMA-UE was used to determine the motor function and degree of muscle synergies present. The FMA-UE consists of four subscales: shoulder, wrist, hand and coordination/speed. A three point scale is used to score movement, with a maximum of 66 points indicating a normal motor function of the upper limb. The internal consistency and validity are excellent [33–37]. The MCID is 6 to 8 points for patients in chronic phase of stroke [38]. The ARAT evaluates arm and hand dexterity on the International Classification of Functioning, Disability and Health (ICF) activity level [35, 39, 40]. The movement is scored on a four point scale with a maximum of 57 points, indicating normal motor function. The MCID is 5.7 points [39]. The reliability and validity are excellent [29, 35–37].

The EuroQoL-5D-5L (EQ-5D) was used to determine the participants’ quality of life using five questions regarding mobility, self-care, usual activities, pain and anxiety/depression. The answers form a five digit number that represents the health state of the participants. This health state can be used to compare stroke patients to the norm values set for the Dutch population [41]. The second part of the EQ-5D comprises of a Visual Analogue Scale (VAS). The participant rated their current health state from 0 (worst) to 100 (best). The MCID is 0.1 for the EQ-5D health state and 8.6 points for the VAS [42]. The EQ-5D has shown reasonable validity and acceptable responsiveness [42–44].

Lastly, questionnaires were used to investigate the participant’s user experience. The Intrinsic Motivation Inventory (IMI), System Usability Scale (SUS) and Dutch-Quebec User Evaluation of Satisfaction with Assistive Technology (D-QUEST). The IMI contains 37 questions regarding interest/enjoyment, perceived competence, effort/importance, pressure/tension, perceived choice, value/ usefulness [45]. The mean score was calculated per subscale, ranging between 1 and 7. Except for the subscale “pressure”, high scores indicate positive effects. For “pressure”, a low score reflects better outcomes. Adequate reliability is reported for the IMI [45]. The SUS consists of ten questions about the perceived usability on a five-point Likert scale [46]. A percentage of the perceived usability was calculated, with 100 being completely satisfied with the usability. If participants gave a score of 70 or more, it was considered that the device was acceptable to use. The SUS has shown to have an excellent reliability, concurrent validity and objective usability [47]. Lastly, the D-QUEST was also used to evaluate the opinion of the participant about the device on a five-point Likert scale [48]. The D-QUEST has shown to be valid, applicable and reliable as measure for user satisfaction regarding (medical) devices [48].

### Measurements

**Fig. 2:**
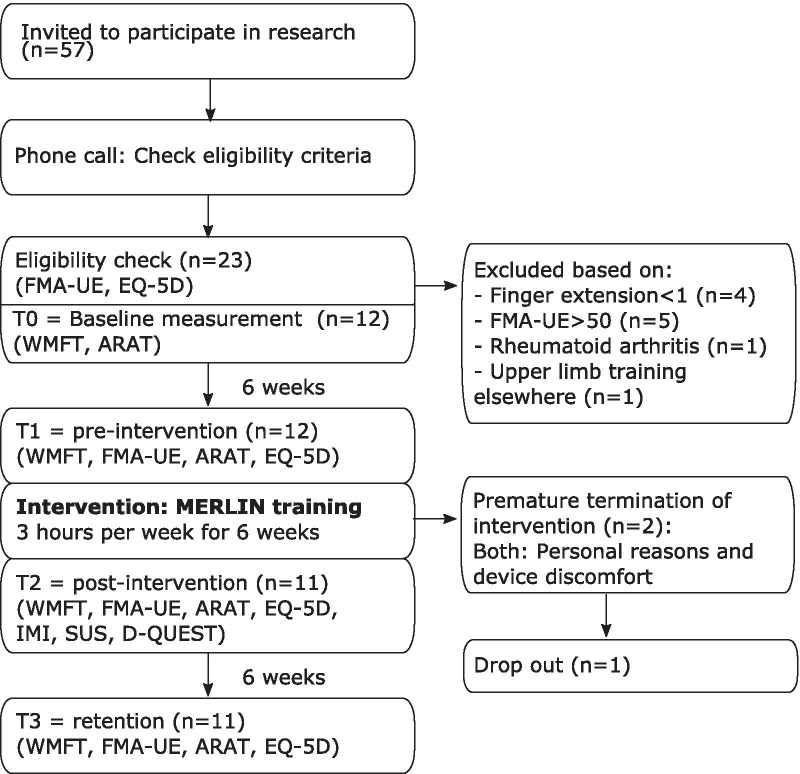
Flow diagram of time points and outcomes.* ARAT* Action Research Arm Test,* D-QUEST* Dutch-Quebec User Evaluation of Satisfaction with Assistive Technology,* EQ-5D* Euro-Quality of Life -5 Dimensions,* FMA-UE* Fugl Meyer Assessment -Upper Extremity,* IMI* Intrinsic Motivation Inventory,* SUS* System Usability Scale,* WMFT* Wolf Motor Function Test. One of the two patients that dropped out was willing to complete the subsequent measurements

All measurements and training sessions took place at the participant’s home. Two independent researchers were trained in performing the tests by a professional occupational therapist with 12 years of experience in treating stroke patients. The independent researchers could not be blinded due to the design of the study, but they were blinded to the participants’ progress in the study. Participants were assessed four times during the study (see Fig. 2). First, the participants were contacted by phone to check the inclusion and exclusion criteria. If they seemed to fulfil the eligibility criteria, the FMA-UE score and EQ-5D health state were assessed during a first visit. If participants were indeed eligible, the meeting would be continued as the first measurement (T0) and the WMFT and ARAT were assessed subsequently. After six weeks, the participants were assessed again (T1). During the period between T0-T1 the participants did not receive any arm/hand training and it was considered as control period. After T1, the participants trained during six weeks with MERLIN. After these six weeks, the arm function tests were repeated (T2). In addition the IMI, SUS and D-QUEST were filled out. To determine the retention of the effects, the arm function tests were repeated six weeks after termination of the training (T3).

### Statistics

Study data were collected and managed using REDCap (Research Electronic Data Capture) hosted at UMCG [49, 50]. REDCap is a secure, web-based software platform designed to support data capture for research. Statistical analyses were performed in IBM SPSS Statistics (version 23). The data were checked for normality using the Shapiro–Wilk test and z-scores for skewness since the sample size was small. If the data was not significantly different from the normal distribution, a repeated measures ANOVA was used. The level of statistical significance was set at alpha < 0.05. If sphericity was violated, the Greenhouse–Geisser correction was applied. A Bonferroni correction was applied during post hoc testing due to multiple measurements. Alpha was set at (0.05/6) = 0.008. Non-parametric Friedman tests were used if the data seemed to be non-normally distributed. Effect sizes were calculated as partial eta squared (η_p_^2^) to identify small(0.02), medium (0.13) or large (0.26) effect sizes [51]. Intention to treat was applied, missing data were replaced by the mean of the previous outcomes of that participant.

### Consequences due to COVID-19 pandemic

Due to COVID-19 pandemic, two of the T2 and seven of the T3 measurements were performed differently. Measurement equipment was delivered at the patient’s home, including a laptop to set up a video connection. The patient performed the arm function tests with assistance of a family member. Via the video connection, two raters observed and individually rated the movement and reached consensus about the scoring. Those protocol changes were approved by a special COVID-19 pandemic task force of the UMCG and were in accordance with the hospital and national regulations (Institutional Review Board, Central Committee on Research Involving Human Subjects (CCMO), National Institute for Public Health and the Environment (RIVM) and UMCG). Three items of the arm function tests could not be performed or had to be adapted. For the FMA-UE, testing reflexes was not possible. However it was not expected that this would change in patients in the chronic phase of stroke, therefore previous scores were used. In the WMFT, the item “weight to box” and “grip strength” are two separate items, independent of the FAS score. The item “weight to box” requires at least two people to assist, which was not feasible and therefore not assessed during online measurements. The digital Biometrics E-link hand dynamometer runs on specific software that was available on the computer of the researcher. Due to the online measurement, it was not possible to use the digital dynamometer and was therefore replaced with an analogue handheld Jamar dynamometer. It has been shown that significant differences exist in the hand grip scores when interchanging the dynamometers [52]. Therefore, the data on hand grip strength were not reliable and not reported.

## Results

Two participants dropped out, one after 3.5 weeks and one after 5 weeks. Reasons for both drop outs were both due to personal reasons and related to discomfort with the device. Their hands were small and the hand grip of the device was too large, this part of the hand grip could not be adjusted. Therefore, they felt uncomfortable using the device. The participant who dropped out after 3.5 weeks did not participate in the T2 and T3 measurements, because she was too weak due to a previous illness. The participant who dropped out after 5 weeks was willing to continue to participate in the post measurement. Participants trained on average 15.2 h (range 2.9–32.8) over the course of six weeks. When discarding the drop outs, the mean training time was 17.4 h (range 5.5–32.8). Five out of the ten participants who completed the training period trained more than the prescribed 18 h.

### Arm/hand function outcomes and quality of life

**Fig. 3:**
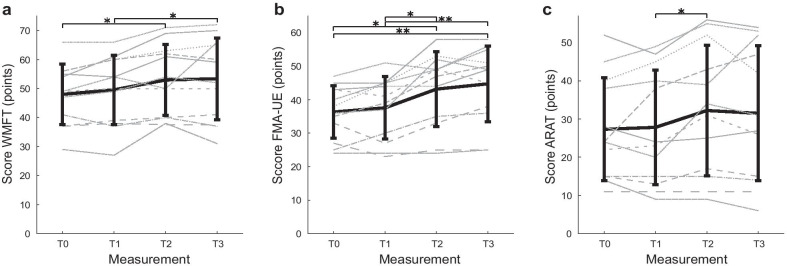
Mean score on arm function measurements over time for the primary outcome Wolf Motor Function (**a**) and secondary outcomes Fugl-Meyer Assessment-Upper Extremity and Action Research Arm Test (**b** and **c**).* ARAT* Action Research Arm Test,* FMA-UE * Fugl-Meyer Assessment-Upper Extremity,* WMFT* Wolf Motor Function Test. T0 = baseline, T1 = 6 weeks after baseline, pre-intervention, T2 = post-intervention, T3 = 6 weeks after intervention. * = p < 0.008, ** = p < 0.001. Thick black line = mean; grey striped/dotted lines: individual data. Vertical bars represent standard deviation, N = 12

Over the course of the study, an improvement in upper limb function was observed (Fig. 3). Regarding the primary outcome, repeated measures ANOVAs revealed significant differences between the measurements for WMFT FAS (F(3,33) = 7.62, p = 0.001, η_p_^2^ = 0.41). No significant differences were found between the measurements for WMFT time (F(3,33) = 0.50, p = 0.689). The post hoc analysis on the WMFT showed a significant difference for T0–T2 (5.0 points) and T1–T3 (3.8 points).

For the secondary outcomes, significant differences were found for the FMA-UE (F(3,33) = 16.51, p < 0.001, η_p_^2^ = 0.6) and ARAT (F(3,33) = 4.04, p = 0.018, η_p_^2^ = 0.27). No significant differences were found between the measurements for EQ-5D health state (F(3,33) = 0.89, p = 0.459) and EQ-5D-VAS (F(3,33) = 2.18, p = 0.109). Post hoc analyses were performed for the arm/hand outcomes (see Appendix: Table 3). No significant differences were found for T0-T1 for all outcomes. In the case of the FMA-UE, significant differences were found for T0–T2 (6.8 points), T0–T3 (8.4 points), T1–T2 (5.6 points) and T1–T3 (7.2 points), indicating that participants significantly improved their arm function during the intervention period. The subscales of the FMA-UE showed that shoulder, elbow, wrist and coordination improved due to training using MERLIN system (see Appendix). A significant difference in the ARAT was found for T1–T2 (4.4 points). The difference between pre-intervention and retention (T1–T3) was 3.7 points. Training effects were retained, since none of the arm function outcomes were significantly different between T2 and T3.

### Device related outcomes

Participants were overall satisfied with the device (Table 2). Eleven participants completed the SUS and D-QUEST questionnaires. The IMI questionnaire was completed by ten participants, one of them was unable to complete it due to cognitive deficits.

**Table 2:** Device related outcomes. (N = 11)

Outcome measure	Mean ± SD
SUS (0–100)	77.27 ± 16.1
D-QUEST device (1–5)	3.65 ± 0.95
D-QUEST service (1–5)	4.41 ± 0.63
D-QUEST total (1–5)	3.90 ± 0.39
IMI* (1–7)	
Interest	5.5 ± 0.50
Competence	5.4 ± 0.67
Effort	6.0 ± 0.35
Pressure	2.2 ± 0.55
Perceived choice	5.6 ± 1.36
Usefulness	6.5 ± 0.17

*D-QUEST* Dutch- Quebec User Evaluation of Satisfaction with assistive Technology, *IMI* intrinsic Motivation Inventory, *SD* standard deviation, *SUS* System Usability Scale, * N = 10, one participant was unable to complete this questionnaire

### Safety

No significant adverse events related to the therapy or the device happened during the clinical research. Reported adverse events were pain in the shoulder (n = 3), neck (n = 2) and hand (n = 1), which were already foreseen and notified to the participants. The hand grip was too small for participants with large hands, causing pressure points on the skin. The thumb support caused pain in four participants. One serious adverse event occurred: one participant was admitted to the hospital during the control period for reasons unrelated to the research activities. Over the course of six weeks, patients needed on average on five occasions assistance via a telephone call, remote assistance with the computer to solve problems with the software or a visit to solve hardware problems. In two out of these five occasions, the patient required an extra visit besides the calibration visits, due to technical problems with the device.

## Discussion

Training with MERLIN for six weeks improved the arm function of patients in the chronic phase of stroke with lasting results six weeks after training cessation. Training at home was feasible and patients were satisfied with the usability of MERLIN system. The participants’ quality of life did not change due to the treatment. Some minor adverse events related to device usage were reported.

The improvements between pre-intervention and retention found in the WMFT (3.8 points; 7.7%) and FMA-UE (7.2 points; 19.1%) were larger than the MCIDs and therefore can be considered clinically meaningful. The improvement on the ARAT (3.7 points; 13.2%) was less than the MCID which could be due to the specific fine motor functions that are assessed in this test which require independent finger control. During the clinical research presented, mass finger and thumb flexion and extension were trained instead of independent finger movements, which may be the reason why participants did not reach clinically meaningful improvements on the ARAT.

The clinical improvement that was shown in this study demonstrates the importance of extended rehabilitation therapy after the subacute phase of stroke. Patients in the chronic phase of stroke often do not receive much therapy due to limited healthcare resources. Our study adds to the growing body of evidence that in the chronic phase of stroke improvements in the upper limb are possible, as was shown in a review by Teasell et al. [7]. The improvement that was observed in our study could either be due to plasticity in the brain or due to counteracting learned non-use. Non-use is the behaviour of choosing to use compensating movements with the non-affected side. Since the affected arm or hand has to be used during training with a non-robotic device, the patient is forced to use the hand. This is similar as during Constrained Induced Movement Therapy, which has shown significant and clinically relevant improvements compared to a control group [53]. It should be considered to change current care protocols to provide more therapy in the chronic phase of stroke. Rehabilitation devices such as MERLIN are a great asset to make this affordable since personnel costs can be reduced. MERLIN includes several functionalities for rehabilitation using less complex technology which is more affordable in comparison to similar devices such as Amadeo and Tyromotion, which are designed for similar therapy including active or passive modes as MERLIN does.

Out of the twelve patients, seven were unable to meet the prescribed training time of 18 h in 6 weeks, although reported motivation was high. Some examples of explanations were: device or software failure, not having enough time due to taking care of children, need for assistance with training. Similar findings regarding difficulties with completing training time were reported in the SCRIPT project in which patients in the chronic phase of stroke trained at home with the non-robotic Saebo Mobile Arm Support and the SCRIPT wrist and hand orthosis [54]. IMI subscale scores and average training time were even lower in comparison to the study presented in this manuscript. A recent review of Chen et al. (2019) regarding telerehabilitation, claims that patient motivation for training is one of the main challenges when patients train unsupervised at home [55]. The advantage of home training is that highly motivated participants have the opportunity to train without time restraints, which is not feasible when they train with a therapist. Participants who trained 18 h or more in our study showed on average more improvement than participants who were unable to fulfil the recommended training time. This finding is in line with a previous review, showing that a dose–response relationship exists for stroke rehabilitation [13]. In the study of Wolf et al. it was also shown that more training could lead to larger improvements in arm function with robotic home training [56]. In their study, larger clinically significant improvements in FMA-UE and ARAT in comparison to the current study were found. Fifty-one patients in the subacute phase of stroke were asked to train at home for 120 h in eight weeks: 80 h with the robotic device and 40 h of functional activities. However, the results showed that patients actually trained 36 h with the device. While training time with the device was recorded, training time for functional activities was kept in a diary which is more prone to overestimation. Therapy compliance was related to the patients’ motivation to improve the upper limb function. Reasons to deviate from the protocol were: lack of motivation to train, responsibilities to work or family or difficulties with using computers. Similar results related to patient motivation were seen in the MERLIN study. Patients who are less motivated or unable to train on their own may benefit more from face-to-face appointments. Therapists could therefore take into consideration if patients are adequately motivated and capable for home rehabilitation without direct supervision. Improvements in software such as adding a large variety in games and levels could improve motivation and patient engagement.

Overall usability of the MERLIN system was rated high (77 out of 100 on SUS). In comparison, in the SCRIPT project the SUS was rated with 69% [54, 57]. According to an elaborated analysis of the SUS by Bangor et al., scores above 70 are deemed good and within the range of acceptable devices [46]. We can therefore conclude that with an average score of 77, the usability of MERLIN is sufficient. There is however room for improvement to increase the usability and prevent the adverse events that were reported in this study. The musculoskeletal problems (pain in shoulder, neck, hand) reported are comparable to other studies that reported device related adverse events [58, 59]. Device improvements are suggested to prevent these adverse events in the future, such as to decrease the height of the device, offer different sizes of the hand grip or make the handgrip more adjustable to customize it for each person and increase the robustness of the system.

The task specific approach that was used in this study is one of the main strengths. The ArmAssist device allows more movements than most training devices that only focus on the shoulder and elbow [60]. The games were entertaining and increased in difficulty which was challenging for the participants. The therapy program was tailored to the participants’ capabilities to challenge the participant as much as possible. Easy communication with the participants and evaluation of their progress was guaranteed by the telecommunication platform. Another strength of home rehabilitation which was shown in this study is the availability of therapy under different circumstances. This was especially shown during the COVID-19 pandemic, participants in the study were able to continue training while usual care was disrupted. Home training could also be a solution for people that live in rural areas, with less resources nearby or difficulties to drive to rehabilitation centers.

On the other hand, some limitations need to be mentioned. First, the study was underpowered due to a lack of suitable patients and two patients that dropped out. Finding suitable patients appeared to be difficult. Many patients who showed interest to participate had a FMA-UE score that was too high for participation. A trial including a larger sample size would be desirable since certain trends that were now observed could yield significant differences in a larger study group. For instance the non-significant trend seen in the quality of life measurements (measured with the EQ-5D) between T1-T2 appeared to be larger than the MCID.

Secondly, we used a within subject design instead of a between subject design using a control group. A control group would have provided stronger evidence of the effectivity in comparison to usual care, but this was not feasible within the time frame of this project, leaving room for future research. The training time that was not met by more than half of the participants has been considered a limitation for the results. Nevertheless, significant improvements in upper limb function were shown with less training time. Lastly, home rehabilitation in its optimal form should require a minimal amount of supervision from a therapist. Additional visits to patients were needed for troubleshooting mostly related to hardware issues, emphasizing that the robustness of the device needs to be improved.

When telerehabilitation with an unactuated robotic device can be provided at minimal costs, this may lead to intensifying upper limb rehabilitation in the chronic phase of stroke. However, many questions regarding home training with (non-)robotic devices are still to be answered. Future research could investigate which underlying factors contribute to not completing the requested amount of training time, since this seems to be a reoccurring problem in telerehabilitation studies.

## Conclusion

With the MERLIN system, telerehabilitation based on serious games combined with an unactuated robotic training device, improved upper limb function in patients in the chronic phase of stroke in a clinically meaningful way. The usability of MERLIN was acceptable and most patients were motivated to train. In contrast to arm function, training did not have an effect on quality of life. Telerehabilitation based on serious games with a non-robotic device at home seems to be effective for highly motivated, moderately affected chronic stroke patients.

## Data Availability

The dataset supporting the conclusions of this article are available via DataverseNL: 10.34894/MBABRA.

## Appendix

See Table 3

**Table 3:** Overview of arm function and quality of life test results using intention to treat analysis (N = 12)

Outcome measures	Mean ± SD				Post hoc analysis
	T0	T1	T2	T3	p-value
WMFT FAS	48.00 ± 10.41	49.58 ± 11.95	52.96 ± 12.24	53.38 ± 14.08	T0–T2 = 0.003 T1–T3 = 0.006
WMFT time	23.65 ± 23.91	24.27 ± 24.97	21.86 ± 19.74	24.24 ± 24.89	
FMA-UE total	36.33 ± 7.83	37.58 ± 9.38	43.17 ± 11.20	44.75 ± 11.33	T0–T2 = 0.004 T0–T3 = < 0.001 T1–T2 = 0.003 T1–T3 = < 0.001
FMA-UE Shoulder	21.5 ± 3.92	21.92 ± 4.27	24.92 ± 5.74	25.92 ± 5.47	T0–T3 = 0.002 T1–T2 = 0.005 T1–T3 = < 0.001
FMA-UE Wrist	5.67 ± 2.93	5.0 ± 2.30	5.46 ± 2.57	6.04 ± 2.72	T1–T3 = 0.004
FMA-UE Hand	6.17 ± 3.27	7.17 ± 3.88	8.38 ± 4.27	8.21 ± 4.35	T0–T2 = 0.003
FMA-UE Coordination/speed	3.0 ± 1.41	3.5 ± 1.24	4.42 ± 1.16	4.58 ± 1.16	T0–T2 = 0.003 T0–T3 = 0.003 T1–T3 = 0.005
ARAT	27.33 ± 13.47	27.83 ± 15.03	32.25 ± 17.09	31.50 ± 17.68	T1–T2 = 0.007
EQ-5D health state	0.70 ± 0.13	0.73 ± 0.13	0.76 ± 0.08	0.74 ± 0.10	
EQ-5D VAS	73.75 ± 18.23	70.08 ± 17.27	79.75 ± 16.23	73.92 ± 15.97	

Repeated measures ANOVA with post hoc analysis. Only significant differences are shown, significant difference = p < 0.008

*ARAT* Action Research Arm Test, *EQ-5D* EurQoL–5 Dimensions, *FAS* Functional Ability Scale, *FMA-UE* Fugl-Meyer Assessment-Upper Extremity, *SD* standard deviation, *VAS* Visual Analogue Scale, *WMFT* Wolf Motor Function Test

## Abbreviations

ARAT: Action Research Arm Test
CCMO: Centrale Commissie Mensgebonden Onderzoek
COPD: Chronic Obstructive Pulmonary Disease
D-QUEST: Dutch- Quebec User Evaluation of Satisfaction with assistive Technology
DoF: Degrees of Freedom
EQ-5D: Euro-Quality of Life -5 Dimensions
FAS: Functional Ability Scale
FMA-UE: Fugl Meyer Assessment-Upper Extremity
Hem: Hemorrhagic
IMI: Intrinsic Motivation Inventory
Isch: Ischemic
MCID: Minimal Clinically Important Difference
MERLIN: HoMEcare aRm rehabiLItatioN
RIVM: Rijksinstituut voor Volksgezondheid en Mileu
SD: Standard deviation
SMART: Sensorimotor Active Rehabilitation Training
SUS: System Usability Scale
T-WREX: Therapy Wilmington Robotic Exoskeleton
UMCG: University Medical Center Groningen
WMFT: Wolf Motor Function Test
